# Decreasing translation error rate in *Escherichia coli* increases protein function

**DOI:** 10.1186/s12896-016-0259-8

**Published:** 2016-03-11

**Authors:** Marina Musa, Miroslav Radman, Anita Krisko

**Affiliations:** Mediterranean Institute for Life Sciences (MedILS), Mestrovicevo setaliste 45, 21000 Split, Croatia

**Keywords:** Protein activity, *rpsL141*, Protein expression systems

## Abstract

**Background:**

Over-expressed native or recombinant proteins are commonly used for industrial and pharmaceutical purposes, as well as for research. Proteins of interest need to be purified in sufficient quantity, quality and specific activity to justify their commercial price and eventual medical use. Proteome quality was previously positively correlated with ribosomal fidelity, but not on a single protein level. Here, we show that decreasing translational error rate increases the activity of single proteins. In order to decrease the amount of enzyme needed for catalysis, we propose an expression system bearing *rpsL141* mutation, which confers high ribosomal fidelity. Using alpha-glucosidase (exo-alpha-1,4-glucosidase) and beta-glucanase (beta-D-glucanase) as examples, we show that proteins purified from *Escherichia coli* bearing *rpsL141* mutation have superior activity compared to those purified from wild type *E. coli*, as well as some commercially available industrial enzymes.

**Results:**

Our results indicate that both alpha-glucosidase and beta-glucanase isolated from *E. coli* bearing *rpsL141* mutation have increased activity compared to those isolated from wild type *E. coli*. Alpha-glucosidase from *rpsL141* background has a higher activity than the purchased enzymes, while beta-glucanase from the same background has a higher activity compared to the beta-glucanase purchased from Sigma, but not compared to the one purchased from Megazyme.

**Conclusion:**

Reduction of the error rate in protein biosynthesis via ribosomal *rpsL141* mutation results in superior functionality of single proteins. We conclude that this is a viable system for expressing proteins with higher activity and that it can be easily scaled up and combined with other expression systems to meet the industrial needs.

## Background

Proteins produced in and isolated from various microorganisms are commonly used for vaccines and other therapeutic purposes, as well as diagnostics, industrial and research purposes. These proteins need to be purified in sufficient quantity and high quality. Thus, we need near perfect proteins, whereas evolution selects efficacy rather than perfection as demonstrated by the ease of selection for high fidelity protein biosynthesis in bacteria, e.g., by streptomycin resistance. Therefore, proteins produced by living cells are far from perfect already during their biosynthesis and folding, and post-synthetically during their intracellular and extracellular maintenance. For instance, we have observed that the proteome of *Deinococcus radiodurans* is five times less oxidized in vivo than the *Escherichia coli* proteome [1]. As the world market for enzymes grows; in 2003 it was estimated at $2.3 billion, and was expected to reach $3.74 billion in 2015 [2, 3], it is important to improve the quality of proteins, in particular therapeutic, and to achieve cost effectiveness of production. This can be done either by optimizing the purification processes, or by optimizing the microorganisms used to overexpress the protein. For example, lowering the growth temperature and the medium pH can help correct folding and improve the activity of alpha-glucosidase expressed in *E. coli* [4]. Overexpressing various protein chaperons in *E. coli* (GroEL/GroES, DnaK/DnaJ/GrpE, ClpB and the small HSPs lbpA/IbpB), alongside desired protein overexpression, has been shown to increase the yield of soluble protein [5]. This approach, however, may impose additional stress on the cell, as it involves overexpressing multiple proteins. An alternative to overexpressing chaperones would be to minimize the need for them by optimizing the translation process, and thus curtailing misfolding.

The prevailing source of protein damage in living cells is oxidative damage. Two main sources of protein oxidation in living cells are reactive oxygen species (ROS) and the intrinsic susceptibility of proteins to oxidative damage. A correlation has been shown between the synthesis of aberrant proteins and their irreversible oxidation (carbonylation), as a result of their increased susceptibility to damage [6]. Analogously, decreasing the error rate during translation via ribosomal mutations reduces protein carbonylation at constant ROS levels [7]. Decreased error rate during translation can be achieved by introducing the *rpsL141* mutation, which carries an Asn for Lys substitution at position 42 of S12 protein of the small ribosomal subunit. *rpsL141* affects the proofreading step in translation i.e. increases the rejection rate of near-cognate tRNAs, thus decreasing amino acid misincorporation [8]. Protein carbonylation level has already been correlated with decreased cellular biosynthetic capacity by Krisko and Radman [7] who showed that both *rpsL141* mutation and chaperone overexpressions (GroEL/ES, Tig, DnaK) increase the single burst size of λ phage in *E. coli*, reflecting increased functionality of the cellular biosynthetic machinery.

As new genes are expressed and screened for protein activity, in order to make their production financially viable, the ability to express proteins in sufficient amount and quality at a reasonable cost becomes a priority [2]. Since it meets the affordability criteria and is easily manipulated and grown on large scale, *E. coli* is the most widely used prokaryotic system for the synthesis of heterologous proteins, including alpha-glucosidase and beta-glucanase [4, 5, 9–11]. We reasoned that reducing the error rate during translation via *rpsL141* mutation, thus reducing proteome susceptibility to oxidative modification, would not only be beneficial for the entire proteome, but would directly and indirectly improve the quality of individual proteins and improve their specific activity.

By expressing alpha-glucosidase and beta-glucanase in *E. coli* bearing the *rpsL141* mutation, we show that it is possible to achieve higher enzyme activity compared to those expressed in the WT background. The advantage of *rpsL141* over chaperone overexpression is that it is a genomic mutation and poses no additional stress, or burden of plasmid maintenance, for the cell. We have, thus, succeeded in reducing the amount of enzyme required to carry out a specific reaction. We believe that this approach can be used for a wide variety of enzymes, as well as in other expression systems, to reduce the amount of enzyme needed for a reaction by improving its activity and additionally, for therapeutic proteins, to reduce their immunogenicity.

## Methods

Gene encoding alpha-glucosidase from *Bacillus stearothermophilus* (pD441_BsAglu) and beta-glucanase from *Aspergillus niger* (pD441_AnBglu) were synthesized and cloned into a pD441 expression vector with a 1 × FLAG peptide at the 3′ end (purchased from DNA 2.0). The constructs were transformed into *E. coli* MG1655 and *E. coli rpsL141*.

Protein extracts were obtained from 1 L of culture, which was prepared by diluting overnight culture 200x in LB with kanamycin and 100 μM isopropyl β-D-1-thiogalactopyranoside (IPTG) and grown to saturation. Cells were harvested by centrifugation and washed with 10 mM PBS, pH 7.4, and the pellet was lysed with 1 mg/mL lysozyme in PBS supplied with protease inhibitor cocktail (Thermo Scientific), at 37 °C for 60 min and cell debris removed by a 20-min centrifugation at 10,000 × g. The remaining supernatant was mixed with Anti-FLAG Agarose Affinity Gel (Sigma), following manufacturer instructions. Cell lysates were incubated with the resin overnight at 4 °C with shaking. Elution was performed with 3 M MgCl_2_ for 30 to 60 min at 4 °C. Eluted protein extract was then concentrated and the buffer was changed to PBS using Amicon centricons with 3 kDa cutoff. Protein concentration was determined using BCA (Pierce) and the product was checked by SDS-PAGE. Purity of extracts was determined using SDS-Page and ImageJ software by plotting each lane on a surface plot and comparing the relative densities of the peaks. Yields were expressed as total protein amount in mg obtained after purification.

For comparison with proteins purified from *E. coli* MG1655 and its congenic *rpsL141* derivative, alpha-glucosidase and beta-glucanase were purchased from Megazyme and Sigma.

Megazyme alpha-glucosidase (E-TSAG) was supplied as an ammonium sulfate suspension, and the buffer was changed to PBS prior to activity measurements using Centricon Centrifugal Units with a 3 kDa cutoff (Amicon). Megazyme beta-glucanase (E-CELAN) was supplied as crystalline enzyme in ammonium sulphate and was reconstituted in PBS prior to activity measurements.

Sigma alpha-glucosidase (G3651) was supplied as lyophilized powder and was reconstituted in PBS prior to activity measurement. Sigma beta-glucanase (49101) was supplied as powder and was reconstituted in PBS prior to activity measurement. Freeze-thaw cycles were minimized by preparing fresh solutions.

The activity of exo-alpha-1,4-glucosidase enzymes produced in the two *E. coli* systems described above was compared to the activity of exo-alpha-1,4-glucosidase purchased from Megazyme (E-TSAGS) and Sigma (G3651). All proteins were transferred into 10 mM PBS pH 7.4 using Centricon Centrifugal Units with a 3 kDa cutoff (Amicon). The concentration of each enzyme was adjusted to 0.5 mg/mL and the activity was measured using the Abnova Alpha-glucosidase Assay kit (KA1608). Briefly, alpha-glucosidase hydrolyzes the terminal, non-reducing 1,4-linked alpha-D-glucose residues with release of alpha-D-glucose. The method utilizes p-nitrophenyl-α-D-glucopyranoside that is hydrolyzed specifically by alpha-glucosidase into a yellow colored product (maximal absorbance at 405 nm). The rate of the reaction is directly proportional to the enzyme activity. Statistical significance was determined using ANOVA plus post hoc, *p*-value < 0.05.

The activity of beta-D-glucanase enzymes produced in the two *E. coli* systems described above was compared to the activity of beta glucanase purchased from Megazyme (E-CELAN) and Sigma (49101). All proteins were transferred into 10 mM PBS with pH 7.4 using Centricon Centrifugal Units with a 3 kDa cutoff. The concentration of each enzyme was adjusted to 0.5 mg/mL and the activity was measured by using the Azo-Barley Glucan Method (Megazyme, S-ABG100). Each protein was incubated with Azo-Barley glucan substrate following manufacturers instructions. The dyed substrate was depolymerized by malt beta-glucanase to fragments which are soluble in the presence of precipitant solution. Upon centrifugation of the precipitant-treated reaction mixture, the absorbance (at 590 nm) of the supernatant solution was directly related to the activity of beta-glucanase in the sample. Statistical significance was determined using ANOVA plus post hoc, *p*-value < 0.05.

Carbonylation was measured as previously described in [7]. In brief, 1 μg protein was bound to Nunc Maxisorp Immunoplates (Sigma) and incubated overnight. Carbonyl groups were derivatized using 2,4-Dinitrophenylhydrazine (Sigma-Aldrich) and labeled using anti-DNP primary antibody. After labeling with HRP-conjugated secondary antibody, absorbance was read at 470 nm.

Statistical analyses of the data were conducted using the GraphPad Prism Software. Since data followed normal distribution, the differences between multiple groups were compared using parametric one-way ANOVA, followed by Tukey’s *post-hoc* test. For all tests significance level was set at *p* < 0.05.

## Results and discussion

In view of the fact that proteome functionality can be improved by ribosomal mutations with decreased translation error rates, we tested whether this is the case for individual proteins by expressing alpha-glucosidase and beta-glucanase in *rpsL141* background and measuring their activity.

**Fig. 1:**
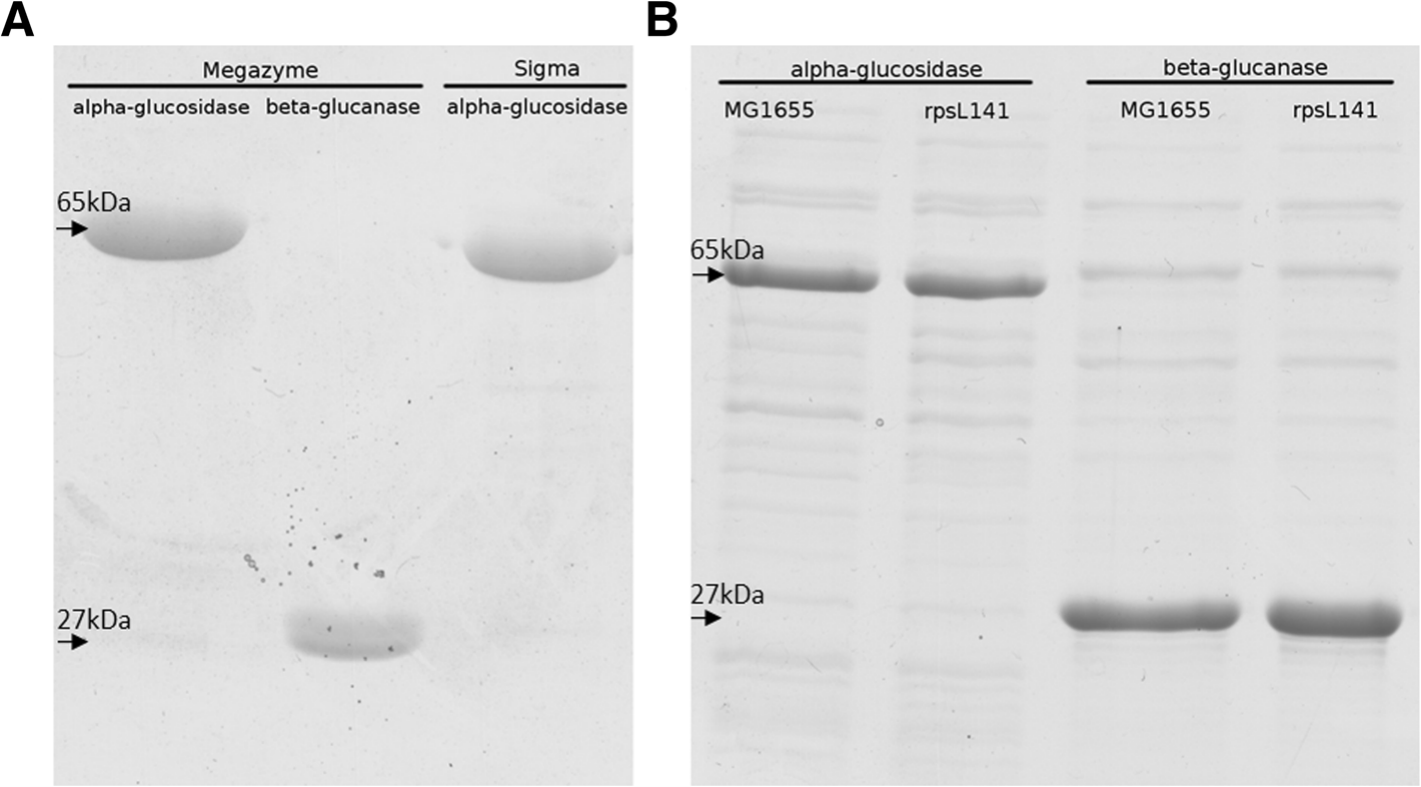
**a** SDS-PAGE of purchased Megazyme and Sigma proteins. **b** SDS-PAGE of alpha-glucosidase and beta-glucanase from MG1655 and rpsL141 background

After purification, alpha-glucosidase and beta-glucanase from both MG1655 and *rpsL141* backgrounds, as well as Megazyme and Sigma enzymes were assayed for their activity. Their purity was checked using SDS-PAGE (Fig. 1.). Alpha-glucosidase shows a prominent band at ≈ 65 kDa and is approximately 86 % pure as estimated by ImageJ software (Fig. 1b). Beta-glucanase displays a band at ≈ 27 kDa, and a less prominent one at ≈ 65 kDa, which are both reported sizes of beta-glucanase [9, 11]; since the ≈ 65 kDa band varies in intensity between samples, we assume the correct band is at ≈ 27 kDa and is ≈ 70 % pure (Fig. 1b). Yields (here expressed as net amount obtained from one extraction from 1 L of bacterial culture) for both enzymes varied from 0.01 mg to 0.5 mg – this however was affected by the method of extraction, as it varied equally between both strains.

**Fig. 2:**
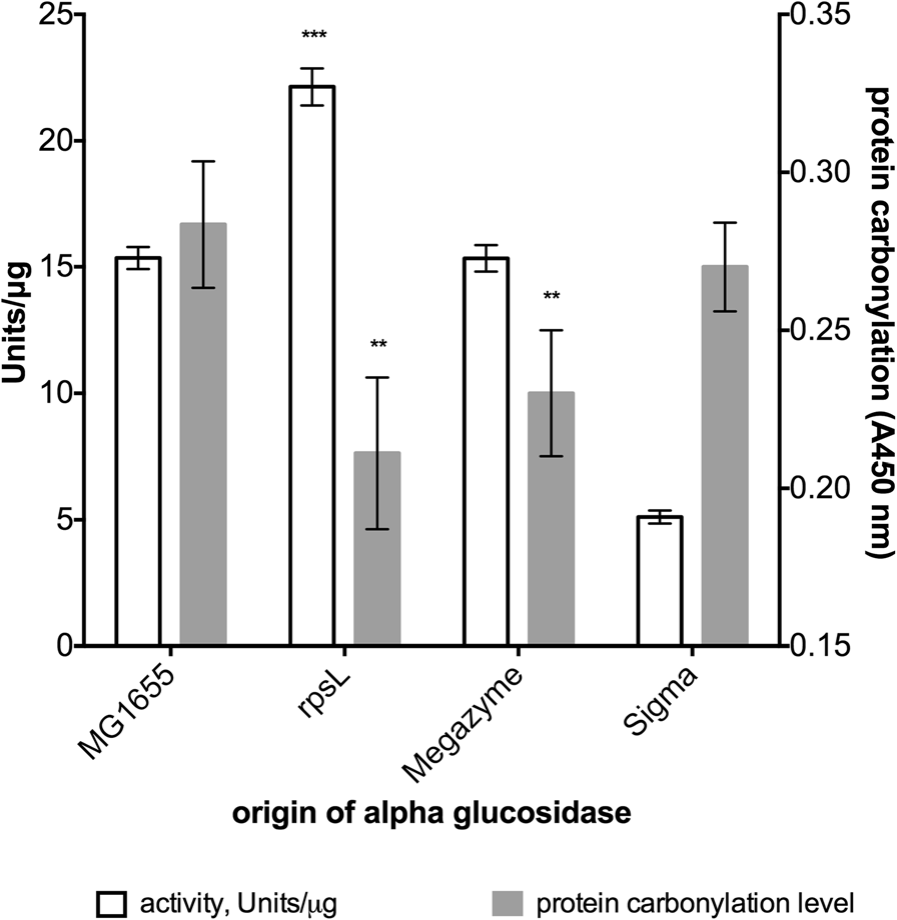
Activity and carbonylation levels of alpha-glucosidase from different backrounds. Figure represents biological and technical replicates, statistical significance was determined using ANOVA plus post hoc, *p*-value < 0.05. *** *p* < 0.001; ** *p* < 0.01

Alpha-glucosidase from *rpsL141* background shows ≈ 30 % increase in activity compared to both alpha-glucosidase from MG1655 background and that purchased from Megazyme. Compared to alpha-glucosidase from Sigma, alpha-glucosidase from the *rpsL141* background has roughly 80 % higher activity (Fig. 2). This suggests that alpha-glucosidase activity can be increased without changing the growth conditions as done in [4]. However, by combining these two approaches it may be possible to obtain alpha-glucosidase with superior activity.

**Fig. 3:**
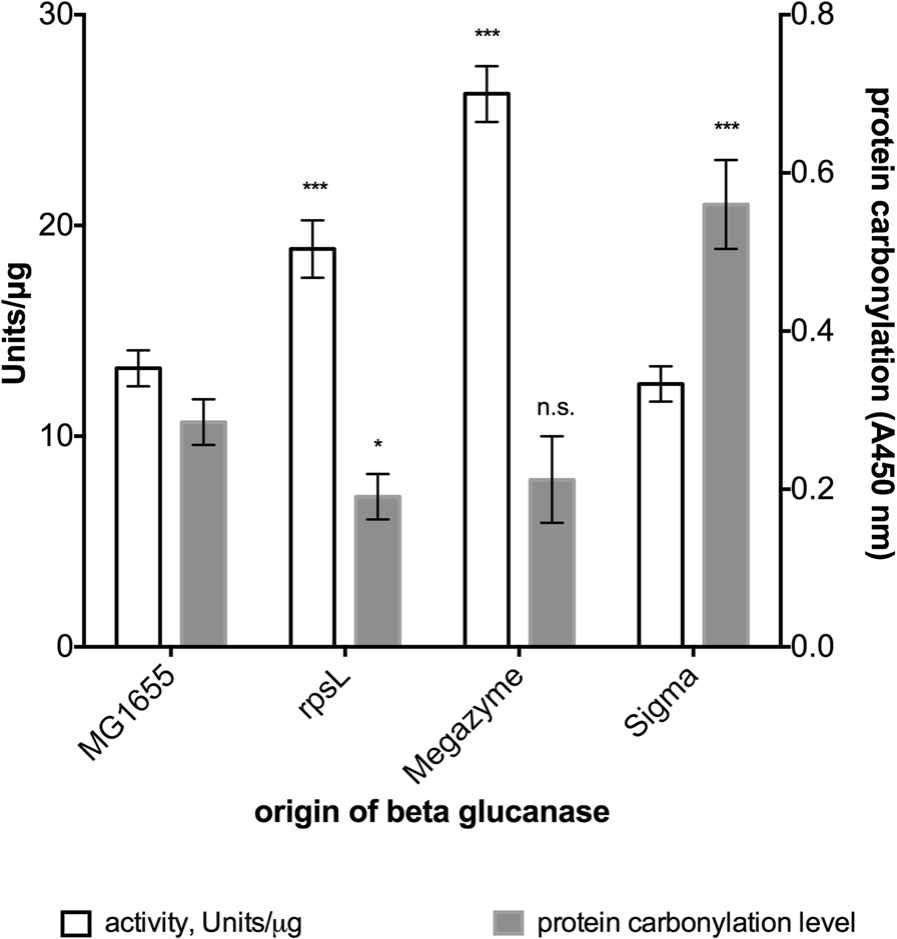
Activity and carbonylation levels of beta-glucanase from different backrounds. Figure represents biological and technical replicates, statistical significance was determined using ANOVA plus post hoc, *p*-value < 0.05. *** *p* < 0.001; ** *p* < 0.01. N.s. stands for not significant

Beta-glucanase activity is also increased when expressed in *rpsL141* background compared to MG1655 and Sigma enzyme, with activity increase of roughly 30 % compared to these two enzymes. However, beta-glucanase from Megazyme showed the highest activity (≈25 % higher than beta-glucanase from *rpsL141* background; Fig. 3).

Since we postulated that this heightened activity is a direct consequence of lower oxidative damage, as a result of favorable folding environment due to lower translation error rates in *rpsL141* mutant, we measured carbonylation levels of all purified and purchased enzymes. Generally, the results demonstrate that heightened activity is paired with lower carbonylation levels (Figs. 2 and 3). This supports our idea that enzyme specific activity can be increased by lowering its oxidative damage, which was achieved by decreasing the translation error rate.

It should be noted that our purified enzymes and the purchased products may not be directly comparable to each other, as we are not familiar with the exact purification process or any modifications made to purchased enzymes that would render them highly active. Furthermore, while our purified enzymes were assayed immediately after purification and quantification, Sigma and Megazyme enzymes were supplied in different buffers and states and were subsequently placed in phosphate buffer (see Materials and Methods). Thus, we used the purchased enzymes simply as a standard against which we test our purified product, and to gauge its potential as a commercial product.

Regardless of these limitations, we believe that this assay supplies sufficient support to the hypothesis that producing enzymes in an *rpsL141-*bearing system, and thus reducing errors in protein synthesis, ultimately leads to enzymes of superior functionality. De Marco and colleagues [5] successfully increased the yield of transgenic proteins from *E. coli*, while Thahn and colleagues [4] increased the enzyme recovery from inclusion bodies. In contrast, we increased not the yield, but the specific activity of purified proteins of interest likely by lowering their susceptibility to oxidative damage, thus presenting a new possibility for obtaining industrial enzymes.

We anticipate that such proteins with improved activity can be obtained using a wide spectrum of purification methods, as well as in different expression systems, and that the purification process can easily be scaled up to meet industrial needs. Since this is a very active field of research, and the need for more sustainable and efficient processes is ever growing, small improvements to the activity of single proteins can be significant for the production process and the industrial or medical usage.

## Conclusions

Our results demonstrate that reducing the translation error rate, and thereby improving proteome quality, ultimately leads to superior functionality of single proteins. Such proteins have a higher activity than those purified from wild type *E. coli*, thus lowering the total amount that would need to be purified and to carry out a given volume of chemical reactions or to provide a given therapeutic effect. We expect that this or a similar expression system bearing the *rpsL141* mutation can be used on a much larger scale, thus reducing the quota for heterologous proteins by improving their functionality.

## Availability of data and materials

Plasmids used in this study are deposited in Addgene: Addgene ID 74220 (pD441_AnBglu); Addgene ID 74221 (pD441_BsAglu).
